# Less inflammatory response in the direct anterior than in the direct lateral approach in patients with femoral neck fractures receiving a total hip arthroplasty: exploratory results from a randomized controlled trial

**DOI:** 10.2340/17453674.2024.41242

**Published:** 2024-08-15

**Authors:** John Magne HOSETH, Otto Schnell HUSBY, Øystein Bjerkestrand LIAN, Tor Åge MYKLEBUST, Tommy Frøseth AAE

**Affiliations:** 1 Department of Orthopaedic Surgery, Health Møre and Romsdal HF, Kristiansund Hospital, Kristiansund; 2 Faculty of Medicine and Health Sciences, NTNU, Trondheim; 3 Department of Neuromedicine and Movement Science, NTNU, Trondheim; 4 The Clinical Research Unit, Health Møre and Romsdal HF, Ålesund; 5 The Cancer Registry of Norway, Oslo, Norway

## Abstract

**Background and purpose:**

It is still debatable which is the least invasive approach to the hip joint in arthroplasty for a femoral neck fracture (FNF). We compared the traditional direct lateral approach (DLA) with the direct anterior approach (DAA) regarding creatine kinase (CK), C-reactive protein (CRP), and hemoglobin (Hb).

**Methods:**

In a randomized controlled trial, 130 elderly patients with dislocated FNFs treated with total hip arthroplasty (THA) were included. CK, CRP, and Hb were measured preoperatively and on postoperative days 1 to 4 and were compared between the DAA and DLA groups using repeated measures mixed-effect models.

**Results:**

The CK level was significantly higher on the 1st postoperative day in the DLA group, 597 U/L (95% confidence interval [CI] 529–666) vs 461 U/L (CI 389–532), estimated mean difference (MD) 136 U/L (CI 38–235). The CRP levels were significantly higher on postoperative days 3 and 4 in the DLA group, 207 mg/L (CI 189–226) vs 161 mg/L (CI 143–180), estimated MD 46 mg/L (CI 19–72) and 162 mg/L (CI 144–181) vs 121 (CI 102–140), estimated MD 41 mg/L (CI 15–68). Blood loss, expressed as difference in Hb, did not differ between the groups.

**Conclusion:**

In an elderly population with FNFs, we found that the DAA, compared with the DLA, results in less CK and CRP increase, but no change in Hb.

The standard treatment for a dislocated femoral neck fracture (FNF) is a hemiarthroplasty (HA) or a total hip arthroplasty (THA). There are 3 conventional routes for accessing the hip joint in the case of an FNF: the posterior approach (PA), the direct lateral approach (DLA), and the direct anterior approach (DAA). The standard posterior approach is not desirable for this patient group due to the high incidence of dislocation reported in several publications [1-3]. The DLA is widely used because of its low dislocation rates [2], but it is associated with muscle weakness due to gluteus medius failure, which may cause limping and pain [4]. The use of the DAA in patients with FNF has demonstrated a substantially lower risk of dislocation compared with the posterior approach [5], and has been introduced as an alternative approach to the hip joint in this patient group [6]. With regard to the invasiveness of the approaches to the hip joint, previous studies on THA in patients with osteoarthritis (OA) have assessed muscle damage, inflammation, and blood loss by comparing differences in serum levels of creatine kinase (CK), C-reactive protein (CRP), and hemoglobin (Hb) [7,8]. The inflammatory response has been little examined in patients with FNF [9,10]. We aimed to compare the effect of DAA, relative to DLA, on CK, CRP, and blood loss on postoperative days 1 to 4, in elderly patients with dislocated FNFs treated with THA.

## Methods

### Trial design

We conducted a prospective randomized controlled trial (RCT) from November 2018 to February 2023 to compare the DAA with the DLA in patients with FNF receiving a THA [11]. This manuscript is based on exploratory results from this RCT, which has several other outcomes. The main results regarding functional outcome will later be reported in another paper. The study followed the CONSORT guidelines.

### Participants

Patients admitted to the orthopedic ward in Kristiansund Hospital, Norway, with an FNF classified as Garden type 3 or 4 were considered for inclusion. To fulfill the inclusion criteria, patients above 50 years of age had to be ambulatory prior to sustaining the fracture and give written consent. The exclusion criteria were infection around the hip, excessive alcohol or substance abuse, pathologic fracture, bedridden patients, multitrauma patients, and patients with dementia or other causes of cognitive impairment.

### Intervention

We compared the DAA with the DLA in a 1:1 ratio. All patients received a THA.

### Outcomes

We compared CK, CRP, and Hb levels between the DAA and the DLA groups. Serum markers were measured at the time of hospitalization prior to surgery and on 4 consecutive days postoperatively during the morning rounds between 7 am and 9 am.

### Sample size

The sample size was based on the primary outcome, which was the Timed Up and Go (TUG) test. We estimated 65 patients for each group, 130 patients in total. We compared our sample size with a previous study comparing inflammatory response (CK and CRP respectively) following the posterolateral approach and DAA in hip OA patients [8]. In that study, the authors calculated a sample size with the power of 90% and an alpha of 5%. Consequently, 23 patients were estimated for each group.

### Randomization and stratification

Patients were randomized to either DAA or DLA using the WebCRF program [12], a secure data collection solution where patients can be randomized, and data can be stored and later retrieved for analysis. After written informed consent was obtained, stratification was performed for the following prognostic factors: (i) pre-fracture place of residence (i.e., home or residential care); (ii) pre-fracture functional status (i.e., using a walking aid or walking independently); (iii) American Society of Anesthesiologists (ASA) Class (i.e., Class I/II or III/IV/V).

The patients were included in the study by the orthopedic resident on duty, while the principal investigator generated the allocation sequence and assigned patients to type of intervention.

There was no blinding of patients, surgeon, or physiotherapist.

### Surgical technique

2 experienced hip surgeons performed all surgeries according to the study protocol. They were both beyond the learning curve of the DAA (> 100 surgeries) [13]. A cemented cross-linked polyethylene acetabular component (Marathon, Dupuy Synthes, Raynham, MA, USA), and a cemented highly polished femoral component (MS-30, Zimmer, Warsaw, IN, USA) were used in all cases. We used a CoCr femoral head (Zimmer) of 32 mm in diameter.

DAA was performed with the patient in supine position. The skin incision was 2 cm distal to and 2 cm lateral to the anterior superior iliac spine. A self-holding retractor between the tensor fasciae lata (TFL) laterally and the sartorius and rectus femoris medially was used. The ascending branch of the lateral circumflex femoral artery was ligated. The anterior part of the capsule was removed. To mobilize the femur, the pubofemoral and ischiofemoral ligaments were released. Components were implanted.

DLA was performed in a lateral decubitus position. A longitudinal incision was made extending 3–5 cm proximal and about 5–8 cm distal to the tip of the greater trochanter. The tendon and muscle fibers of the gluteus medius were split at the midway point between the anterior and posterior extent of the muscle. After implantation, the capsule was closed with interrupted sutures. The gluteus medius muscle was reattached with osteosutures.

For both approaches, the fascia was closed with running sutures, the subcutaneous tissue with non-running sutures, and the skin with non-absorbable running sutures.

### Serum markers of muscle damage, inflammation, and blood loss

CK, CRP, and Hb were used as serum markers as a surrogate measure of muscle damage, inflammation, and blood loss respectively. CK is mostly found in skeletal muscle, and is a marker of skeletal muscle breakdown [14]. The CK reference range is 30–135 U/L for females and 55–170 U/L for males, varying with age, race, and muscle mass [15]. CRP is a common biomarker of infection and inflammation, normally < 5 mg/L. In uneventful orthopedic surgery, the peak level occurs on the 2nd or 3rd postoperative day, and reflects the extent of the total inflammatory response including fall, fracture, and the surgical trauma [16]. Blood loss was measured as the difference in serum Hb (g/dL) concentration from preoperatively to days 1, 2, 3, and 4 postoperatively. The 1st 4 days after surgery were chosen because of the corresponding half-life of CK and CRP at 12 and 19 hours respectively (14, 16). We corrected for transfusions in the statistical analysis.

### Statistics

Descriptive statistics on demographic and clinical parameters are presented using mean and standard deviation (SD). To analyze differences in serum CK, CRP, and Hb concentrations over time, we estimated repeated-measures mixed-effect models (RMMEM) with random intercepts for each patient to facilitate the longitudinal structure of the data. We estimated separate models for each of the 3 outcomes, controlling for baseline measurement as a covariate in the model. For blood loss we also included the number of transfused units of blood. In addition, all models included study group, time, and interaction between study group and time as covariates. From the estimated models, we predicted marginal means, and the corresponding 95% confidence intervals (CI), for each combination of time and study group. We assessed pairwise differences using the Wald test. Lastly, we used logistic regression to estimate odds ratio (OR) for receiving blood transfusion. P values < 0.05 were considered statistically significant. All analyses were performed using IBM SPSS version 29 (IBM Corp, Armonk, NY, USA) and STATA version 18.0 (StataCorp LLC, College Station, TX, USA).

### Ethics, registration, data sharing, funding, use of AI, and disclosures

The regional ethics committee (REK) approved the randomized controlled trial (ID 2018/935). The study was registered in clinicaltrials.gov (ID NCT03695497, Protocol ID 2018/935). Data sharing is possible upon request, which requires fulfillment of law regulations before distribution to foreign countries. The authors received no funding for this work and have no conflicts of interest. AI was not used. Complete disclosure of interest forms according to ICMJE are available on the article page, doi: 10.2340/17453674.2024.41242

## Results

**Figure 1:**
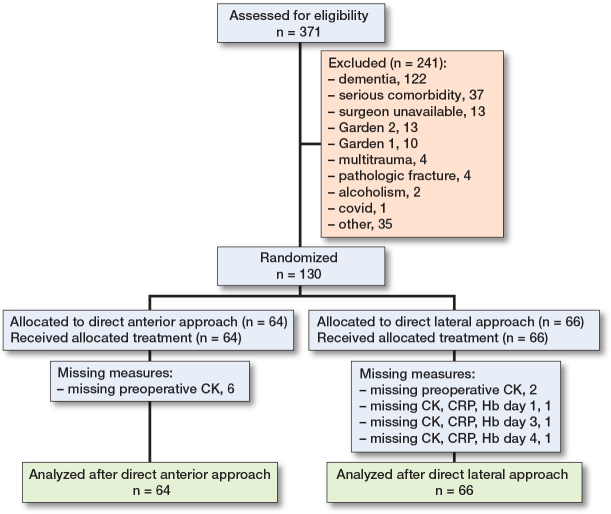
Consort flowchart. Analysis of creatine kinase (CK), C-reactive protein (CRP), and hemoglobin (HB) preoperatively and on days 1, 2, 3, and 4 postoperatively.

During the inclusion period, 371 patients with FNF were admitted to the orthopedic ward. 130 patients were included, while 241 patients did not fulfill the inclusion criteria (Figure 1). 122 patients were excluded due to dementia or other causes of cognitive impairment, while 37 patients were excluded due to serious comorbidity. 13 patients were not included because the study surgeons were absent, and 23 patients were excluded due to non-displaced femoral neck fractures. Of the 130 included patients, 64 patients were allocated to DAA and 66 were allocated to DLA (Figure 1). 29 patients were lost to follow-up during the study period. No patients were lost to follow-up the 1st 4 postoperative days; however, 11 patients had missing values. In the DAA group 6 patients had missing preoperative CK. In the DLA group 2 patients had missing preoperative CK, 1 patient had missing CK, CRP, and Hb on day 1, 1 patient had missing CK, CRP, and Hb on day 3, and 1 patient had missing CK, CRP, and Hb on day 4. Demographic data included age, sex, body mass index (BMI), and ASA grade, which were similar between the 2 groups (Table 1). Of all patients, 21% were operated on < 12 hours after the injury, 38% 12–24 hours after the injury, 32% 24–48 hours after the injury, and 9% > 48 hours after the injury.

**Table 1:** Demographics and preoperative data of the study population. Values are count unless otherwise specified

Factor	DLA	DAA
Females, n (%)	41 (62)	42 (66)
Age, mean (SD)	79.1 (1.2)	78.1 (1.2)
BMI, mean (SD)	23.8 (0.5)	25.0 (0.6)
ASA class		
1	2	0
2	16	17
3	46	43
4	2	4
Time to surgery in hours		
0–12	15	12
12–24	25	24
24–48	23	19
> 48	3	9
Pre-fracture walking aid	19	17
Admitted from residential care	3	2
General anesthesia, n (%)	9 (14)	8 (13)

DAA: direct anterior approach

DLA: direct lateral approach

BMI: body mass index

ASA: American Society of Anesthesiologists

The DAA group had an average surgery time of 79 minutes (SD 12), which was shorter than the DLA group (84 minutes [SD 14], P < 0.02).

### CK

**Figure 2:**
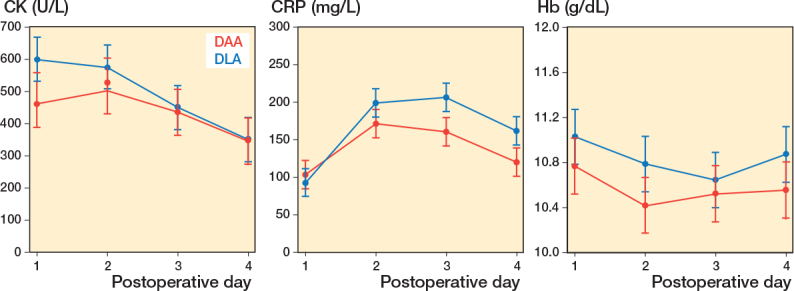
Differences in creatine kinase (CK), C-reactive protein (CRP), and hemoglobin (HB) after RMMEM analysis, presenting 95% confidence interval for each group on the different days following surgery.

There was no difference in preoperative CK levels between DAA 146 U/L (SD 195) and DLA 142 U/L (SD 198), MD 4 U/L (SD 196). The CK level was significantly higher for DLA on the 1st postoperative day, 597 U/L (CI 529–666) vs 461 U/L (CI 389–532), estimated MD 136 U/L (CI 38–235), but not the following days (Table 2), (Figure 2).

**Table 2:** Mean values and differences in CK, CRP, and Hb levels after repeated-measures mixed-effect models (RMMEM) analysis

Factor	DLA (CI)	DAA (CI)	Difference (CI)
CK (U/L)			
Preop. (SD)	142 (198)	146 (195)	–4 (196)
Day 1	597 (529–666)	461 (389–532)	136 (38 to 235)
Day 2	574 (506–643)	503 (431–574)	71 (–27 to 170)
Day 3	449 (381–518)	436 (365–508)	13 (–86 to 112)
Day 4	350 (282–419)	345 (274–417)	5 (–94 to 104)
CRP (mg/L)			
Preop. (SD)	9 (16)	23 (38)	–14 (29)
Day 1	93 (75–112)	104 (85–123)	–11 (–37 to 16)
Day 2	198 (180–217)	171 (153–190)	27 (0.9 to 54)
Day 3	207 (189–226)	161 (143–180)	46 (19 to 72)
Day 4	162 (144–181)	121 (102–140)	41 (15 to 68)
Hb (g/dL)			
Preop. (SD)	12.5 (1.5)	12.9 (1.9)	–0.4 (1.7)
Day 1	11.0 (10.8–11.3)	10.8 (10.5–11.0)	0.2 (–0.1 to 0.6)
Day 2	10.8 (10.5–11.0)	10.4 (10.2–10.7)	0.4 (0.02 to 0.7)
Day 3	10.6 (10.4–10.9)	10.5 (10.3–10.8)	0.1 (–0.2 to 0.5)
Day 4	10.9 (10.6–11.1)	10.6 (10.3–10.9)	0.3 (–0.04 to 0.7)

CK: creatine kinase

CRP: C-reactive protein

Hb: hemoglobin

DAA: direct anterior approach

DLA: direct lateral approach

CI: confidence interval

SD: standard deviation

### CRP

The preoperative CRP levels were higher for the DAA, 23 mg/L (SD 38), than for DLA, 9 mg/L (SD 16), MD 14 mg/L (SD 29). The CRP levels were significantly higher for the DLA on postoperative day 3 and on day 4: 207 mg/L (CI 189–226) vs 161 mg/L (CI 143–180), estimated MD 46 mg/L (CI 19–72) and 162 mg/L (CI 144–181) vs 121 (CI 102–140), estimated MD 41 mg/L (CI 15–68) (Table 2, Figure 2).

### Blood loss

There was no difference in the preoperative Hb value between DAA, 12.9 g/dL (SD 1.9), and DLA, 12.5 g/dL (SD 1.5), MD 0.4 g/dL (SD 0.4). On postoperative day 2, the groups differed somewhat: the DLA had the highest Hb concentration, 10.8 g/dL (CI 10.5–11.0) vs 10.4 g/dL (CI 10.2–10.7), estimated MD 0.4 g/dL (CI 0.02–0.7), but the difference was not considered statistically or clinically significant (Table 2, Figure 2). 47 (36%) patients received transfusions, 22 of 64 (34%) in the DAA group, and 25 of 66 (38%) patients in the DLA group. The groups did not differ in the odds of receiving a transfusion (OR 1.2, CI 0.6–2.4). The DAA group received 60 units of blood (1 unit corresponds to 300 mL), with an average of 2.7 units per patient transfused. The DLA group received 57 units of blood (2.3 units per transfused patient), i.e., no statistically significant difference.

## Discussion

The study aimed to compare the effect of DAA, relative to DLA, on CK, CRP, and blood loss on postoperative days 1 to 4, in elderly patients with dislocated FNFs treated with THA. We showed that postoperative tissue damage, as measured by a change in serum CK, and inflammation, as measured by a change in CRP, were lower in the DAA group compared with the DLA group. This corresponds well with the technique of the DAA where no muscles are detached, i.e., a muscle-sparing approach. Our findings suggest less surgical trauma in patients operated on with DAA than with DLA during the early postoperative days.

CK was significantly elevated in both groups. The average difference in CK between the groups on day 1 after surgery was almost 3 times the normal CK value. This could possibly have an effect on postoperative renal function in elderly frail patients. Our findings suggest that the DAA in patients with FNF produces less inflammation than the DLA in terms of the CRP levels measured postoperatively. Perioperative CRP levels may correlate with postoperative mortality in patients undergoing hip fracture surgery [17].

Most studies to date assessing tissue damage and inflammation following THA in DAA and DLA are in patients with OA, not FNF [7,18,19]. For OA patients, it is difficult to claim that one approach is superior to the others regarding CK and CRP levels, as different studies show conflicting results. Contrary to our findings, Mjaaland et al. found that the CK levels were significantly higher after DAA than after DLA in OA patients [7]. They suggested that stretching of the muscles would release more CK than cutting the muscle, as per gluteus medius detachment in the DLA. Patients with FNF usually have less muscle mass than the younger OA population. Hence, they could be less sensitive to the stretching of muscles in DAA. Ugland et al. [20] compared the DLA with the Watson Jones approach (WJA) in patients with FNF who received an HA. They found an increased CK level postoperatively after WJA, meaning that their CK results point in a different direction than ours, although their population was comparable and the WJA also is considered a muscle-sparing approach.

There have been very few studies of a population with FNF receiving a THA in regard to tissue damage, inflammation, and blood loss. A recent RCT [21] compared early clinical efficacy of the SuperPath approach with the DLA in THA for FNF. Similar to our findings, they found a significant rise in CK in favor of the minimally invasive SuperPath, but they could not demonstrate a difference in CRP between the 2 approaches. Although they reported repeated measurements of CK and CRP, they did not include an RMMEM analysis, in contrast to our study.

The slightly higher Hb concentration in the DLA group compared with the DAA group did not reach statistically or clinically significant difference at any time. The DAA may be associated with greater blood loss than the DLA in patients with OA [7]. This can be explained by the surgical access to the hip joint in the DAA. The intermuscular layer between the TFL on the lateral side and the sartorius and the rectus femoris on the medial side is shielded by the ascending branches of the lateral circumflex artery, which vary in number. After ligature or diathermia, these arteries could rebleed postoperatively.

The DAA can be a challenging approach to master, and without proper training it can lead to unnecessary complications [13]. The learning curve for the DAA is said to be approximately 100 cases [22]; in our opinion the minimum amount of cases needed is 50–100 cases annually.

### Strengths and limitations

*Strengths.* The main strengths of our RCT are, first, that there was an adequate sample size with no differences in patient demographics due to stratification and, second, that all surgeries were performed by 2 surgeons experienced with both approaches.

*Limitations.* It is uncertain whether less tissue damage and inflammation measured by CK and CRP translates into a better outcome regarding pain and mobility. Although we tried to create equal groups by stratifying on certain factors, there may have been many unknown patient characteristics that could have played a role as confounding factors, such as different medications and comorbidities. Controlling for different medications could pose a challenge due to polypharmacy. We did not control for preoperative creatinine or creatinine clearance in the RMMEM analysis, but the influence of renal function on CK levels seems to have been negligible [23]. Our study population had a mean BMI reflecting normal weight. In the DAA, entering the joint is easier in slim or lean patients than in obese or muscular patients. This may be reflected in the lower concentrations of CRP levels postoperatively in the DAA group than in the DLA group.

Measuring blood loss is complicated, and measuring a decrease in Hb values is not entirely accurate when we know that Hb is a measure of concentration influenced by intravenous fluid administration and diuresis in the pre-, intra-, and postoperative periods. There was no set limit to how often the patients were transfused; they were given blood when indicated. They received blood transfusion when postoperative anemia was symptomatic and interfering with mobilization. This threshold was usually lower for patients with coronary heart disease and other comorbidities.

## Conclusion

In our study, we found lower postoperative CK and CRP levels after DAA than after DLA, which indicate less muscle damage and inflammation. We found no difference in blood loss between the groups.

From a clinical perspective, our findings suggest that DAA may reduce tissue damage and inflammation. Further studies need to confirm whether these potential advantages in the immediate postoperative period can enhance mobilization and reduce morbidity and mortality in the fracture population.

## Notes

JMH: original idea, study setup, interpretation of data, drafting of manuscript. OSH: study setup, interpretation of data, revision of manuscript. ØBL: revision of manuscript. TAM: interpretation of data, revision of manuscript. TFA: interpretation of data, revision of manuscript.

Handling co-editors: Cecilia Rogmark and Robin Christensen

*Acta* thanks Volker Thomas Christian Otten and Bjarke Viberg for help with peer review of this manuscript.
